# Boosting Antitumor Response by Costimulatory Strategies Driven to 4-1BB and OX40 T-cell Receptors

**DOI:** 10.3389/fcell.2021.692982

**Published:** 2021-06-30

**Authors:** Daniele E. Mascarelli, Rhubia S. M. Rosa, Jessica M. Toscaro, Isadora F. Semionatto, Luciana P. Ruas, Carolinne T. Fogagnolo, Gabriel C. Lima, Marcio C. Bajgelman

**Affiliations:** ^1^Brazilian Biosciences National Laboratory (LNBio), Brazilian Center for Research in Energy and Materials (CNPEM), Campinas, Brazil; ^2^Faculty of Pharmaceutical Sciences, University of Campinas, Campinas, Brazil; ^3^Medical School, University of Campinas (UNICAMP), Campinas, Brazil; ^4^Medical School of Ribeirão Preto (FMRP), University of São Paulo, Ribeirão Preto, Brazil; ^5^Pro Rectory of Graduation, University of São Paulo, São Paulo, Brazil

**Keywords:** immunotherapy, T cell costimulation, TNFR, 4-1BB, OX40, cancer therapy, aptamers, agonistic antibody

## Abstract

Immunotherapy explores several strategies to enhance the host immune system’s ability to detect and eliminate cancer cells. The use of antibodies that block immunological checkpoints, such as anti–programed death 1/programed death 1 ligand and cytotoxic T-lymphocyte–associated protein 4, is widely recognized to generate a long-lasting antitumor immune response in several types of cancer. Evidence indicates that the elimination of tumors by T cells is the key for tumor control. It is well known that costimulatory and coinhibitory pathways are critical regulators in the activation of T cells. Besides blocking checkpoints inhibitors, the agonistic signaling on costimulatory molecules also plays an important role in T-cell activation and antitumor response. Therefore, molecules driven to costimulatory pathways constitute promising targets in cancer therapy. The costimulation of tumor necrosis factor superfamily receptors on lymphocytes surface may transduce signals that control the survival, proliferation, differentiation, and effector functions of these immune cells. Among the members of the tumor necrosis factor receptor superfamily, there are 4-1BB and OX40. Several clinical studies have been carried out targeting these molecules, with agonist monoclonal antibodies, and preclinical studies exploring their ligands and other experimental approaches. In this review, we discuss functional aspects of 4-1BB and OX40 costimulation, as well as the progress of its application in immunotherapies.

## Introduction

Immunotherapy explores the host immune system to enhance antitumor response. The inhibition of immunological checkpoints, on T cells, such as anti–programed death 1 (PD-1)/programed death 1 ligand (PD-L1) and cytotoxic T-lymphocyte–associated protein 4 (CTLA-4), has been shown to generate long-lasting antitumor immune responses in cancer therapy. However, this approach is effective in only 30% of patients because of mechanisms of tumor resistance (Chen and Mellman, 2013; Swart et al., 2016). There are several signaling mechanisms that may drive T-cell phenotype switching the balance between immunotolerance and surveillance.

The tumor necrosis factor receptor superfamily (TNFRSF) encodes T-cell costimulatory receptors that may regulate survival, proliferation, differentiation, and effector functions of immune cells, being a potential target for immunotherapy (Calderhead et al., 1993; Godfrey et al., 1994; Wilcox et al., 2002; Zhang et al., 2010; Melero et al., 2013).

Early experiments with activated T cells have described the cell surface 4-1BB receptor (Kwon and Weissman, 1989; Schwarz et al., 1993). The 4-1BB receptor, also known as TNFRSF9 or CD137, is a 24-kDa protein located on chromosome 1 p36 (Pollok et al., 1993; Hurtado et al., 1997), which encodes 255 amino acids harboring 64% homology to the murine sequence (Alderson et al., 1994; Zhou et al., 1995; Chin et al., 2018). The 4-1BB receptor is expressed on activated T and B cells, monocytes, macrophages, dendritic cells (DCs), regulatory T cells (Tregs), and natural killer (NK) cells (Pollok et al., 1993; Langstein et al., 1998; Melero et al., 1998; Laderach et al., 2002; Kim et al., 2008; Schoenbrunn et al., 2012). The 4-1BB receptor harbors two cytoplasmic domains that can bind to the TNFR-associated factor (TRAF) in T cells. The mammal TRAF may exhibit conserved C-terminal domains (Bouwmeester et al., 2004; Xu et al., 2004), and CD137 can interact with TRAF1, TRAF2, and TRAF3 (Jang et al., 1998). The interaction between CD137, TRAF1, and TRAF2 activates different signaling cascades such as nuclear factor κB (NF-κB), MAPK (protein kinase activated by mitogen) (Shuford et al., 1997; Arch and Thompson, 1998; Kim et al., 1998), ERK (kinase regulated by extracellular signal), and JNK (Jun N-terminal kinase) (Rothe et al., 1995; Froelich et al., 1996; Arch and Thompson, 1998; Jang et al., 1998; Saoulli et al., 1998; Cannons et al., 2001; Song et al., 2004; Sabbagh et al., 2008). The activation of the NF-κB pathway upregulates survival genes such as Bcl-XL and Bfl-1, downregulates proapoptotic molecules such as BIM, and transmits signals that stimulate cell division (Hockenbery et al., 1990; Lee et al., 2002; Bukczynski et al., 2003). Moreover, the 4-1BB/4-1BB ligand (4-1BBL) signaling triggers biochemical signals that increase T_H_1 cytokines, such as interleukin 6 (IL-6), IL-8, TNF, IL-12, and interferon γ (IFN-γ); suppress T_H_2 cytokines; potentiate activation, survival, proliferation, and cytotoxicity of T cells; increase IL-2 production; and provoke the maturation of DCs (Shuford et al., 1997; Kim et al., 1998; Laderach et al., 2002; Lee et al., 2002; Wen et al., 2002; Lippert et al., 2008; Fröhlich et al., 2020).

The expression of 4-1BB may be experimentally induced in human and murine T cells, using different mitogenic agents such as PMA (phorbol 12-myristate 13-acetate), phytohemagglutinin, anti-CD3, lipopolysaccharide, and IL-1β (Pollok et al., 1995; Zhou et al., 1995; Tan et al., 2000). The receptor interacts with 4-1BBL, on the surface of activated antigen-presenting cells (APCs), B cells, macrophages, and other myeloid-derived cells (Goodwin et al., 1993; Pollok et al., 1994; DeBenedette et al., 1997). The expression of 4-1BBL also was observed in cancer cells (Salih et al., 2000). The 4-1BB costimulation of T cells does not require additional CD28 signaling when a strong engagement of T-cell receptors (TCRs) occurs, producing high levels of IL-2 and enhancing T_H_1 response and cytotoxic T-cell activity (Shuford et al., 1997; Saoulli et al., 1998; Takahashi et al., 1999; Cannons et al., 2000; Dawicki et al., 2004).

Another T-cell costimulatory receptor that belongs to the TNFRSF family is the OX40 receptor, also known as CD134, TNFRSF4, and ACT35. The OX40 receptor and its ligand (OX40L, TNFSF4, CD252) were first described in mice and harbor 63% homology to that of human (Paterson et al., 1987; Mallett et al., 1990; Godfrey et al., 1994). The human OX40 gene is located in on chromosome 1p36, encodes 277 amino acids, a 29-kDa protein (Latza et al., 1994).

The OX40 is expressed in activated CD4^+^ and CD8^+^ T cells, Tregs, T follicular helper cells, NK cells, and neutrophils (Mallett et al., 1990; Godfrey et al., 1994; Melero et al., 1998; Bansal-Pakala et al., 2004; Baumann et al., 2004; Vu et al., 2007; Zaini et al., 2007; Tahiliani et al., 2017). The antigenic stimulation of the TCR by major histocompatibility complex molecules upregulates OX40 expression on the surface of T cells, which may be potentiated by a CD28–CD80 signaling (Walker et al., 1999; Rogers et al., 2001). The OX40 peak expression is seen from 24 to 72 h after the TCR activation (Rogers et al., 2001; Song et al., 2008; Sadler et al., 2014). Once expressed, OX40 can bind to OX40L, which is mainly expressed on APCs (Linton et al., 2003; Jenkins et al., 2007; Karulf et al., 2010). The OX40/OX40L interaction triggers a signaling cascade, similar to 4-1BB/4-1BBL, inducing transcriptional changes to modulate the immune response, such as T-cell proliferation and survival. The immunomodulatory functions associated with OX40, such as the PKB pathway, promote the inhibition of cellular apoptosis, as well as increase the signaling by TCR antigenic stimuli (Song et al., 2004). The OX40 stimulation activates NF-κB pathway, which indirectly increases expression of apoptosis-suppressing proteins prolonging cell survival, and the activated T-cell nuclear factor (NFAT) pathway that leads to an increase in the synthesis of cytokines such as IL-2, IL-4, IL-5, and IFN-γ (So and Croft, 2007). Although, some data suggest the importance of OX40/OX40L signaling for primary and memory T_H_2 response (Salek-Ardakani et al., 2003; Jenkins et al., 2007; Pattarini et al., 2017), there are also evidences that OX40 costimulation plays an important role in the T_H_1 response (Weinberg et al., 1999; De Smedt et al., 2002; Gajdasik et al., 2020). Therefore, it was observed that OX40 costimulation may enhance both a T_H_1 and a T_H_2 response without supporting the role for switching polarization of CD4^+^ T cells (De Smedt et al., 2002).

In this review, functional aspects of 4-1BB and OX4 will be discussed, as well as the progress of its application in immunotherapies.

## Costimulation of 4-1BB and OX40 Receptors Enhances T-Cell Activity and Potentiates Antitumor Response

**FIGURE 1:**
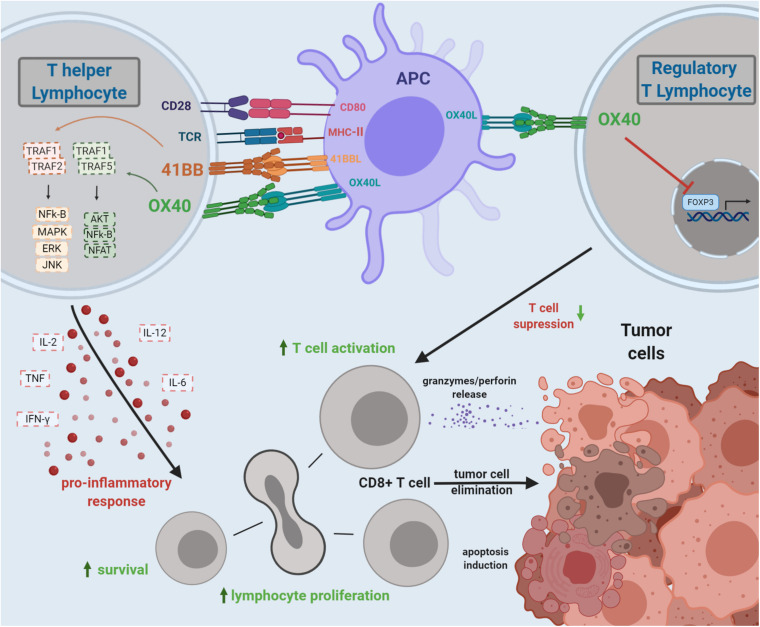
Costimulation by 4-1BB and OX40 TCRs and the overall effects on antitumor T-cell immunity. 4-1BB and OX40 bind to their ligands, triggering a signaling cascade leading to T-cell activation and expansion of cytotoxic CD8^+^ T lymphocytes. Costimulation of OX40 may also inhibit the FOXP3 transcription factor on CD4^+^ T cells, impairing the Treg function, diminishing tumor immunosuppression, and boosting the antitumor immune response. The 4-1BB/4-1BBL signaling triggers biochemical signals that increase T_H_1 cytokines and suppress T_H_2 cytokines; potentiate activation, survival, proliferation, and cytotoxicity of T cells; and provoke the maturation of dendritic cells. The OX40/OX40L interaction triggers a signaling cascade, similar to 4-1BB/4-1BBL, inducing transcriptional changes to modulate the immune response. The OX40 costimulation may promote T-cell proliferation and survival. The agonistic signaling transduced by OX40 on Treg may impair FOXP3 expression, enhancing antitumor response.

Both 4-1BB and OX40 costimulatory signaling on T cells are reported to boost antitumor immune responses (Figure 1).

The costimulatory 4-1BB signaling induces clonal expansion, proliferation, cytokine secretion, and long-term T-cell survival (Wen et al., 2002). The 4-1BB costimulation improves the mitochondrial function of T cells, leading to greater longevity and memory capacity of these cells (Menk et al., 2018). Although 4-1BB costimulation transduces a robust costimulatory signal, mainly acting on CD8^+^ T cells (Habib-Agahi et al., 2007), mice that constitutively express 4-1BB on CD4^+^ T cells exhibited heightened and sustained proliferative activity and enhancement of T-cell priming, driving T_H_1 immune responses, increasing the number of tumor-infiltrating lymphocytes (TILs) in tumor masses, augmenting IFN-γ production by T-cell population, mediating tumor suppression, and prolonging mice survival (Kim et al., 2003). This persistence of T cells is crucial for the success of chimeric antigenic receptor (CAR) T-cell therapy. The CAR has an extracellular domain that recognizes a target antigen, transducing a signaling to an intracellular domain that boosts T-cell activation. This technology is being used mainly in hematological cancers, with promising results (Kalos et al., 2011; Porter et al., 2011). CAR T cells may employ a cytoplasmic domain of 4-1BB molecule to enhance the antitumor efficacy, the so-called third generation of CART cells. The 4-1BB intracellular signaling domain provides a second costimulatory signal that makes the CAR T-cells more effective and long lasting (van der Stegen et al., 2015).

Similar to 4-1BB/4-1BBL, the OX40/OX40L pathway also triggers strong costimulatory signaling on CD4^+^ and CD8^+^ T cells, although this effect is more prominent in CD4^+^ T cells (Xiao et al., 2004). The OX40 costimulatory signaling promotes the activation, expansion, proliferation, differentiation, proinflammatory cytokine production, supporting survival of T lymphocytes, and tumor regression (Rogers et al., 2001; Linch et al., 2016; Peng et al., 2019; Kuang et al., 2020). It was demonstrated that high expression of OX40 in the tumor-infiltrating immune cells in small cell lung cancer induced increased levels of IFN-γ expression and favorable prognosis in patients (Massarelli et al., 2019). The OX40 costimulatory signaling on exhausted CD8^+^ T cells could rescue their proliferative potential and cytokine production (Buchan et al., 2015).

In addition, agonistic OX40 signaling in Tregs may impair the expression of FOXP3, which is a master key to regulate the Treg-immunosuppressive phenotype (So and Croft, 2007; Vu et al., 2007; Piconese et al., 2008). As Tregs may antagonize antitumor response inhibiting T-cell proliferation (Liu et al., 2009; Xu et al., 2010; Chang et al., 2012), the costimulation of OX40 may inhibit the immunosuppressive activity of Tregs and also the conversion of effector T (Teff) CD4^+^ T cells into a regulatory T-cell phenotype (So and Croft, 2007; Piconese et al., 2008; Kitamura et al., 2009). The role of agonistic 4-1BB signaling on Tregs is controversial. Although most studies demonstrated 4-1BB agonistic signaling enhanced the immunosurveillance activity, there are other reports that describes immunosuppressive activity, as expanding Tregs (Irie et al., 2007; Zhang et al., 2007; Lubrano di Ricco et al., 2020).

## Enhancing Antitumor Immunity by Agonistic Antibodies Driven to 4-1BB and OX40 T-Cell Costimulation

The agonistic 4-1BB or OX40 costimulation by monoclonal antibodies (mAbs) is widely explored in cancer immunotherapy (Wilcox et al., 2002; Vinay and Kwon, 2014). There are several clinical trials exploring the therapeutic benefit of mAbs (Table 1). The TNFRSF-targeted costimulatory strategy may be used in combination with chemotherapy and radiotherapy, improving antitumor response (Shi and Siemann, 2006; Ju et al., 2008). 4-1BB agonist antibodies promote increased T-cell proliferation and survival. These antibodies may also activate NK cells (Klatzmann et al., 1984; Lin et al., 2008; Rajasekaran et al., 2015; Makkouk et al., 2016). The binding of an agonistic mAb induces 4-1BB cell surface receptor internalization, which may trigger signaling from endosomatic compartments, as the polyubiquitination of K63 to recruit TRAF2 and starting the T-cell activation cascade (Martinez-Forero et al., 2013). The agonistic 4-1BB antibody may act on depletion of Treg cells, and this stimulus seems to be associated with its FCγR engagement and antibody isotype. It was observed the immunoglobulin G1 (IgG1) mAb isotype induced an enhanced CD8 T-cell costimulation in an established solid tumor microenvironment. The IgG2a isotype has shown intratumoral Treg depletion and optimal antitumor activity in preclinical model (Buchan et al., 2018). The main 4-1BB agonist antibodies used in the clinic are utomilumab (PF-05082566) and urelumab (BMS-663513). Both mAbs are already being used in cancer clinical trials. Whereas the IgG2 utomilumab is safe with relatively low efficacy, the IgG4 urelumab has a great antitumor efficacy, but causes liver damage (Segal et al., 2014; Gopal et al., 2017; Tolcher et al., 2017). In addition, urelumab can induce a cluster of 4-1BBL–dependent receptors, unlike utomilumab (Chin et al., 2018). The preliminary results of urelumab trials in 2008 demonstrated efficacy against cancers in advanced stages. However, the treatment with mAb induced liver damage, interrupting the clinical trials. Toxicity is suggested as a consequence of FcγRIIB-mediated CD8^+^ T-cell activation in the liver, once FcγRIIB is expressed on liver sinusoidal endothelial cells and Kupffer cells (Qi et al., 2019). Urelumab has been evaluated as monotherapy or in combination with other drugs, such as rituximab, cetuximab, elotuzumab, and nivolumab, at lower doses, with no damage to the liver. When associated with rituximab, in the treatment of patients with relapsing or refractory non-Hodgkin lymphomas, the urelumab has demonstrated safety, tolerability, and improvement of the host immune response. Utomilumab promoted a long-lasting response and reduced toxicity in patients with lymphomas. This mAb is being extensively tested in combination with other immunotherapies for different tumors, such as non-small cell lung cancer, kidney, head, and neck cancer (Tolcher et al., 2017; Gopal et al., 2020).

**TABLE 1:** Clinical protocols in progress using costimulatory anti–4-1BB and anti-OX40 monoclonal antibodies.

mAbs	Target	Phase	Tumor	Combination	Protocol
Utomilumab	41BB	**II**	HER2 + Breast cancer	Avehnnab (anti-PD-Ll)	NCT03414658
Utomilumab	41BB	**I**	HER2 + Breast cancer	Trasluzumab (anti-HER2)	NCT03364348
Urelumab	41BB	**I**	Glioblastoma	Nivolumab (anti-PDl)	NCT02658981
AGEN2373	41BB	**I**	Advanced Solid Tumor	–	NCT04121676
MEDI0562	OX40	**I**	Head/Neck squamous cell carcinoma Melanoma	–	NCT03336606
MEDI6469	OX40	**lb**	Head/Neck squamous cell carcinoma	Surgical resection	NCT02274155
MEDI6469	OX40	**I/Ib**	Metastatic colorectal cancer	Radiofrequency ablation	NCT02559024
INBRX-106	OX40	**I**	Locally Advanced/Metastatic tumor	Pembrolizumab	NCT04198766
PF04518600	OX40	**I/II**	Acute myeloid leukemia	Avelumab (anti-PD-Ll) Azacitidine	NCT03390296
PF04518600	OX40	**II**	Metastatic kidney cancer	Axitinib (TK inhibitor)	NCT03092856
PF04518600	OX40	**II**	Triple negative breast cancer	Avelumab (anti-PD-Ll)	NCT03971409
PF-04518600	OX40	**I/II**	Advanced malignancies	Avelumab (antiPD-Ll) Radiation	NCT03217747
INCAGN01949	OX40	**I/II**	Pancreatic cancer	VLP-encapsulated TLR9	NCT04387071
SL-279252	OX40	**I**	Advanced solid tumor/Lymphoma	Fc anti-PDl	NCT03894618

Innovative strategies based on bispecific antibodies also may be used to overcome systemic toxicity of agonistic 4-1BB, targeting the costimulatory activity to tumor site. A bispecific 4-1BB/HER-2 antibody was engineered to bind HER-2–positive tumor cells and to costimulate T cells. This bispecific antibody was shown to inhibit tumor growth in humanized mice model (Hinner et al., 2019).

In addition to the costimulatory 4-1BB mAbs, several studies have also demonstrated the antitumor effect of OX40 agonist antibodies. Mice treated with OX40 mAbs accumulated CD4^+^ T cells and augmented CD4^+^ T-cell survival and developing memory T cells (Cannons et al., 2001; Tolcher et al., 2017). The OX40 agonistic antibody may inhibit immune tolerance (Gramaglia et al., 2000; Maxwell et al., 2000).

The use of OX40 agonistic antibody in preclinical models has shown tumor regression in sarcomas, melanoma, colon carcinoma, and glioma (Kjaergaard et al., 2000; Weinberg et al., 2000; Andarini et al., 2004). In several preclinical and clinical models, OX40 agonist antibodies induce tumor regression due to their ability to prevent the suppression of antitumor immune responses mediated by Tregs. The OX40 costimulation may inhibit Treg activity in three different ways: (i) inhibiting the activity of natural Treg or even the conversion of T cells to Treg phenotype, due to impairing of FOXP3 expression (So and Croft, 2007; Vu et al., 2007; Song et al., 2008); (ii) reducing the suppressive activity by increasing the production of IL-2 and other cytokines (Valzasina et al., 2005); and (iii) depleting intratumoral Treg cells in an FcγR-dependent manner (Bulliard et al., 2014).

A phase I clinical trial in patients, with incurable metastatic carcinoma, lymphoma, or sarcoma (NCT01644968), using 9B12, a murine agonistic anti-human OX40 mAb, demonstrated that OX40 mAb treatment induced proliferation of CD4^+^ and CD8^+^ T cells and NK cells, enhanced production of IFN-γ by CD8^+^ T cells, boosted T- and B-cell antitumor reactivity, and increased memory T cells (Curti et al., 2013). The MOXR 0916, also known as pogalizumab, is a humanized agonistic IgG antibody specific for OX40 that has immunostimulatory and antineoplastic activities. Pogalizumab binds and selectively activates OX40. The activation of OX40 promotes the proliferation of Teff lymphocytes and inhibits the activity of Treg cells in the presence of tumor antigens (Infante et al., 2016).

The combination of agonistic OX40 mAb to the PDL1 inhibitor atezolizumab is being tested in patients who have advanced solid neoplasms (NCT02410512). Recently, Kuang et al. (2020) reported the development of a new anti-OX40 antibody, the IBI101. This mAb promotes both FcγR-dependent and independent agonistic activities. The combination of IBI101 and anti–PD-L1 has shown a better inhibition of tumor growth in mice model, when compared to the combination of pogalizumab and anti–PD-L1 (Kuang et al., 2020).

There are several clinical trials exploring the application of costimulatory anti-OX40 mAbs as adjuvants in other therapies, as anti-OX40 combined to chemotherapy and radiotherapy (NCT01303705), anti-OX40 combined to other immunotherapy and chemotherapy (NCT03390296), and anti-OX40 combined to radiotherapy (NCT01862900, NCT02559024), among others.

## Agonistic Signaling Strategies Mediated by the TNFSF Ligands 4-1BBL and OX40L Potentiate T Cell–Mediated Antitumor Response

As reported above, agonistic TNFRSF-driven antibodies may exhibit toxicity associated with the systemic use, as the expression of TNFRs is ubiquitous, and, consequently, the action of these antibodies is not specific to tumor microenvironment (Ascierto et al., 2010). Therefore, a challenge faced by researchers is to induce a tumor-specific effect, which may reduce toxicity. Back in 2009, the group of Shirwan has developed a murine recombinant 4-1BBL molecule fused to streptavidin (SA–4-1BBL) that exhibited less toxicity when compared to agonistic antibody, lacking FcγR activation (Schabowsky et al., 2009). SA–4-1BBL forms tetramers and oligomers in soluble form, inducing a powerful T-cell costimulatory activity. Further studies have shown this molecule to be safe and effective in murine model of cervical cancer (Sharma et al., 2010). SA–4-1BBL could induce an efficient CD4^+^ T-cell activation while blocking the development of CD4^+^ Tregs, increasing the Teff-to-Treg ratio (Curti et al., 2013). Prophylactic administration of SA–4-1BBL to tumor-challenged mice was shown to prevent tumor growth, and this was dependent on CD4^+^ T cell, NK, and IFN-γ production (Barsoumian et al., 2016).

Murine studies with 4-1BBL have shown that NK cells are activated and may respond to the antitumor activity due to 4-1BBL administration (Houot et al., 2009). Dowell et al. have demonstrated that stimulation of human PBMC, both from healthy donors and ovarian carcinoma patients with soluble 4-1BBL and IL-2, promoted expansion of CD56^+^ NK cells (Dowell et al., 2012). Cancer cells transduced with 4-1BBL promote expansion of cytotoxic T cells (Sharma et al., 2010; Barsoumian et al., 2016), as well as NK cells (Gong et al., 2010). It was also observed that stimulation of human NK cells with recombinant 4-1BBL (rh4-1BB) in combination to anti–HER-2 therapy (trastuzumab) enhanced antitumor cytotoxicity in gastric cancer cells (Misumi et al., 2018).

The 4-1BBL has also been used in cell-based strategies to stimulate TILs. Gene-pulsed DCs with 4-1BBL enhanced IFN-γ production and T-cell activation (Youlin et al., 2013). A lipid-based nanoparticle harboring 4-1BBL is being used for T-cell costimulation, in clinical trials for cancer therapy (NCT03323398, NCT03739931).

As seen for 4-1BBL, the OX40L-expressing cells also stimulate antitumor response. Intratumoral administration of OX40L-expressing DCs promoted the generation of tumor-specific CD4^+^ and NK T cells, contributing to impaired tumor growth (Zaini et al., 2007). Modification of tumor cells with an adenovirus encoding OX40L promoted an efficient T_H_1 immune response associated with cytotoxic T lymphocytes (Andarini et al., 2004).

As observed for combinations of costimulatory mAbs, the OX40L and 4-1BBL dual costimulation has been shown to be effective in reducing tumor growth and enhances mainly the CD8^+^ T-cell response (Weinberg et al., 2000; Cuadros et al., 2005; Lee et al., 2006; Gray et al., 2008; Redmond et al., 2009; Manrique-Rincón et al., 2017). We and others have shown that the synergy between OX40/OX40L and 4-1BB/4-1BBL also contributes to enhance antitumor immune response (Lee et al., 2004, 2006; Munks et al., 2004; Manrique-Rincón et al., 2017; Fu et al., 2020). Indeed, our group has previously developed tumor-derived vaccines using B16 melanoma cells line, transduced with 4-1BBL and/or OX40L. We have shown that combination of these vaccines increased antitumor T cell–mediated cytotoxicity, reduction of Tregs, and contributing to tumor rejection *in vivo*, besides generating a protective effect on rechallenged animals (Manrique-Rincón et al., 2017). Moreover, extracellular vesicles (EVs) secreted by 4-1BBL and OX40L B16 vaccines were shown to induce T-cell proliferation and inhibit the generation of inducible Treg *in vitro* (Semionatto et al., 2020). These findings highlight the exploration of tumor-derived EVs as a potential tool for immunotherapy (Zaini et al., 2007).

## Oligonucleotide-Derived Aptamers May Be Engineered as Costimulatory Molecules to Enhance Antitumor Immunity

Aptamers are small molecules of single-stranded RNA or DNA oligonucleotides that may exhibit high affinity and selectivity for targets (Ellington and Szostak, 1990; Tuerk and Gold, 1990). These molecules exhibit similar properties to antibodies and may have some advantages: (i) aptamers are chemically synthesized and (ii) exhibit high tissue permeability and cell internalization due to its reduced size; (iii) aptamers usually exhibit low toxicity and immunogenicity, and (iv) aptamers may be inactivated by an antidote (Ellington and Szostak, 1990; Bompiani et al., 2012; Cheng et al., 2013; Bouvier-Müller and Ducongé, 2018).

Several aptamer-based therapeutic application has been explored for cancer (Morita et al., 2018; Maimaitiyiming et al., 2019).

Preclinical studies have demonstrated an equivalent or even superior functionality of these oligonucleotides compared to mAb molecules, as well as a decrease of the commonly observed side effects of mAbs (Miller and Chapman, 2001; Dollins et al., 2008; McNamara et al., 2008; Pastor et al., 2011; Pratico et al., 2013; Schrand et al., 2014, 2015; Rajagopalan et al., 2017). Aptamers may be employed as antagonist, agonist, and delivery tools (Pastor et al., 2018). The first aptamer driven to tumor immunomodulation was an antagonist CTLA-4 aptamer, and then, a number of new aptamers with immunomodulatory activity have been proposed (Santulli-Marotto et al., 2003).

As T-cell surface receptors such as TNFRSF are activated because of cross-link, multivalent aptamer models have been explored to lead T-cell activation and costimulation (Dollins et al., 2008; McNamara et al., 2008; Pratico et al., 2013). The possibility of using synthetic linkers of different size and composition was shown. This multimerization strategy allowed to generate functional aptamer molecules that could costimulate 4-1BB and OX40 receptors in T cells that would address improvement of the antitumor immune response (Dollins et al., 2008; McNamara et al., 2008). These studies have shown that treatment with aptamers induces a costimulatory effect on tumor environment comparable to the treatment with the corresponding mAb (Dollins et al., 2008; McNamara et al., 2008; Pratico et al., 2013).

Considering the side effects of systemic administration of mAb 4-1BB (Niu et al., 2007) due to plasticity of engineering, aptamers have been investigated to overcome toxicity, opening a new field of research based on bispecific aptamers. The aim of these approaches is to decrease the off-target effect by driving the costimulatory effect specifically on target cells (Pastor et al., 2011; Schrand et al., 2014, 2015). A first study involving a proof of concept of these aptamers developed a bifunctional 4-1BB–prostate-specific membrane antigen (PSMA) conjugate. The PSMA is a membrane antigen highly expressed in some prostate cells. In this way, the 4-1BB portion had a T-cell costimulatory activity, whereas the PSMA portion could drive the molecule to PSMA-expressing tumor cells. The study showed that systemic administrations of bispecific aptamer were able to inhibit tumor growth with the administration of a 10-fold lower dose without occurrence of side effects (Pastor et al., 2011).

Another technological approach that has been pursued is the generation of chimeric aptamer to vehiculate siRNA to target cells. Aptamers may bind to cell surface receptors to deliver siRNA to the target cell. This approach is target specific and may reduce possible off-target effects, improving the therapeutic index of the use of siRNA as drug treatment (Berezhnoy et al., 2012). A functional study has shown that 4-1BB aptamer was conjugated to a siRNA that negatively regulate the mTOR pathway, decreasing the generation of memory cells in treated animals (Berezhnoy et al., 2014).

Despite the variety of preclinical studies involving aptamer molecules for 4-1BB and OX40 and the positive results achieved, these molecules have not been tested in clinical studies yet. The use of aptamer molecules as a therapeutic strategy is considered recent when compared to mAbs, and it has raised attention in the scientific community, proving its safety profile with the first drug approved for clinical use (Ng et al., 2006) and with other molecules being pursued for clinical study in the field of tumor immunology (Zboralski et al., 2017; Steurer et al., 2019).

## Conclusion

The 4-1BB and OX40 TNFRSF receptors are associated with several signaling that contribute to potentiate T-cell antitumor activity. Agonistic antibodies are widely used in clinical trials and represent the most explored TNFSF-based costimulatory strategies. The development of agonistic oligonucleotide-derived aptamers driven to TNFSFR elements is also an interesting alternative to antibodies because of the simplicity of production through chemical synthesis and engineering possibilities to generate bispecific aptamers or even vehiculated targeted cargos to enhance T-cell function. Signal transduction at TNFSFR receptors can also be mediated by approaches using 4-1BBL or OX40L ligands. These ligands may be expressed in antitumor vaccines or even used in soluble form, exhibiting therapeutic effect. The costimulatory potential of TNFSF molecules is also explored for T-cell engineering, such as chimeric receptors that encode 4-1BB intracellular domains, which are known to play an important role in the costimulation and maintenance of the activated lymphocyte phenotype. Therapeutic strategies that employ 4-1BB and OX40 costimulation work to switch the balance of the immune system toward immunosurveillance. These strategies can also be associated with immunological checkpoint inhibitors, which target the inhibition of immunosuppression mechanisms favoring the detection and elimination of tumor cells.

## Author Contributions

All authors listed have made a substantial, direct and intellectual contribution to the work, and approved it for publication.

## Conflict of Interest

The authors declare that the research was conducted in the absence of any commercial or financial relationships that could be construed as a potential conflict of interest.
